# *Salmonella* Infection Causes Hyperglycemia for Decreased GLP-1 Content by Enteroendocrine L Cells Pyroptosis in Pigs

**DOI:** 10.3390/ijms23031272

**Published:** 2022-01-24

**Authors:** Yibo Zong, Wenjing Chen, Yongsen Zhao, Xiaoyi Suo, Xiaojing Yang

**Affiliations:** MOE Joint Key Laboratory of Animal Physiology and Biochemistry Nanjing Agricultural University, Nanjing 210095, China; 2019207008@njau.edu.cn (Y.Z.); 2019107028@njau.edu.cn (W.C.); T2021120@njau.edu.cn (Y.Z.); 2020207065@njau.edu.cn (X.S.)

**Keywords:** *Salmonella*, hyperglycemia, enteroendocrine L cell, GLP-1, pyroptosis, pig

## Abstract

Inflammatory responses have been shown to induce hyperglycemia, yet the underlying mechanism is still largely unclear. GLP-1 is an important intestinal hormone for regulating glucose homeostasis; however, few studies have investigated the influence of digestive tract *Salmonella* infection on enteroendocrine L cell secretions. In this study, we established a model of *Salmonella*-infected piglets by oral gavage in order to analyze the effects of *Salmonella* infection on enteroendocrine L cell function. Furthermore, in vitro lipopolysaccharide (LPS) was administered to STC-1 cells to clarify its direct effect on GLP-1 secretion. The results showed that significantly increased blood glucose in the group of *Salmonella*-infected piglets was observed, and *Salmonella* infection decreased blood GLP-1 content. Then, ileal epithelium damage was observed by histological detection, and this was further verified by TUNEL staining. We identified activation of TLR signaling demonstrating up-regulated expressions of TLR4 and nuclear factor-kappa B (NF-ΚB). Furthermore, it was shown that *Salmonella* induced pyroptosis of enteroendocrine L cells and enhanced the secretion of IL-1β through augmenting gene and protein expressions of NOD-like receptor protein 3 (NLRP3), apoptosis-associated speck-like protein containing a carboxyl-terminal CARD (ASC), Caspase 1, and gasdermin D (GSDMD). Meanwhile, in vitro LPS treatment induced the pyroptosis of STC-1 cells and reduced the secretion of GLP-1. Altogether, the results demonstrated that *Salmonella* infection can reduce secretion of GLP-1 by inducing pyroptosis of intestinal L cells, which may eventually result in hyperglycemia. The results provided evidence for the cause of hyperglycemia induced by inflammation and shed new light on glucose homeostasis regulation.

## 1. Introduction

Normal human physiology is dependent on tight control over blood glucose levels regulated by various hormones. Imbalanced body glucose homeostasis may result in many clinical symptoms. Hyperglycemia has been shown to induce numerous pathological changes in cell injury, including oxidative stress and increased endothelial cell apoptosis [1,2]. Proinflammatory responses could conceivably create a chronic inflammatory state, resulting in glucose metabolism disorder and potentially triggering immune dysfunction [3]. In recent years, it has been recognized that chronic inflammation may contribute to high blood glucose and disordered insulin levels via the endocrine effects of inflammatory molecules [4,5]. It has been reported that LPS-induced factors released by macrophages include inflammatory mediators such as IL-6, TNF, and interferons that contribute to the development of glucose metabolism disorder [6,7,8]. However, the influence of inflammatory effects on hormones involved with the regulation of glucose metabolism remains largely unclear.

Glucagon-like peptide 1 (GLP-1) is secreted in response to nutrients in the intestinal lumen, such as carbohydrates, fats, and proteins. GLP-1 promotes insulin synthesis, enhances postprandial insulin secretion from pancreatic β cells, and inhibits glucagon secretion, thereby lowering postprandial blood glucose levels [9,10]. Based on these important features, native GLP-1-based therapies have been used for type 2 diabetes mellitus (T2D). GLP-1 is mainly secreted by the gut enteroendocrine L cells, which show an increase in density towards the distal ileum and the proximal colon. Previous studies have found that *Salmonella* infection of mice drastically alters the intestinal metabolome, such as the metabolism of steroid and eicosanoid classes of hormones [11]. However, the effect of intestinal pathogenic bacterium on intestinal GLP-1 production is still incompletely understood.

Pyroptosis, another form of programmed necrosis, was long deemed as caspase-1-mediated inflammatory cell death in response to certain pathogenic bacteria [12]. Inflammasome is a large multiprotein complex that plays critical roles in host defenses against microbial pathogens during the process of pyroptosis [13]. Activation of inflammasomes often requires a priming signal induced by Toll-like receptors. NLRP3 inflammasome, a member of the NOD-like receptor (NLR) family, is assembled in response to a broad range of microbial pathogens. Gasdermin D (GSDMD), a large gasdermin family, is inserted into the membrane, forming pores and inducing pyroptosis [14]. Pyroptosis, which is induced by inflammation in many diseases, can exacerbate tissue damage [15]. Whether *Salmonella* infection influences intestinal epithelial pyroptosis and finally leads to GLP-1 production is still unknown.

Therefore, in the present study, piglets exposed to intestinal *Salmonella* infection were employed. This study aimed to investigate whether *Salmonella* infection impairs the secretion of GLP-1 by enteroendocrine L cells and what the possible underlying mechanism involved is. The results will help to clarify glucose metabolism disorders induced by environmental *Salmonella* infection and shed new light on immunometabolism research.

## 2. Results

### 2.1. Effects of Salmonella Infection on Plasma Parameters of Piglets

As shown in Table 1, the level of TNFα in plasma was significantly higher in the *Salmonella* infection group compared with the control group, while the level of IL-10 in plasma was significantly lower in the *Salmonella* infection group compared with that of the control group. Compared with the control group, the *Salmonella* group piglets presented significantly higher levels of fasting blood glucose (FBG) content along with lower insulin in plasma. Meanwhile, plasma GLP-1 concentrations were dramatically lower in the *Salmonella* infection group compared with the control group. Furthermore, HOMA-IR in the *Salmonella* group was dramatically higher compared with that in the control piglets group.

### 2.2. Effects of Salmonella Infection on Ileum Inflammation

**Figure 1:**
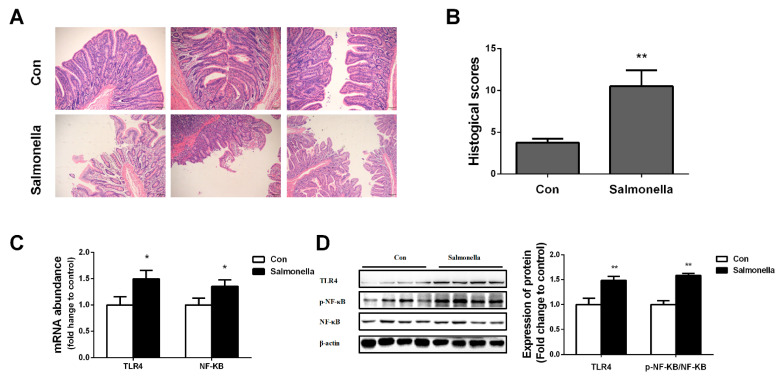
Effects of *Salmonella* on ileum inflammation. (**A**) ileal morphology; (**B**) histological scores; (**C**) TLR4 and NF-ΚB mRNA expressions in ileum; (**D**) TLR4 and p-NF-ΚB/ NF-ΚB protein expressions in ileum. Values are mean ± SEM, *n* = 4/group. (*) means *p* < 0.05, (**) means *p* < 0.01.

Histological analysis revealed that *Salmonella*-challenged pigs demonstrated more ileal villi fracture, epithelial detachment, and increased infiltration of inflammatory cells in the mucosal layer of ileum compared with control group pigs (Figure 1A,B). In addition, we also observed that challenging *Salmonella* significantly enhanced the mRNA expressions of TLR4 and NF-ΚB (Figure 1C); protein expressions of TLR4 and p-NF-ΚB/NF-ΚB were also higher in the *Salmonella* infection group compared with the control group (Figure 1D).

### 2.3. Effects of Salmonella Infection on GLP-1 Secretion in Ileum

**Figure 2:**
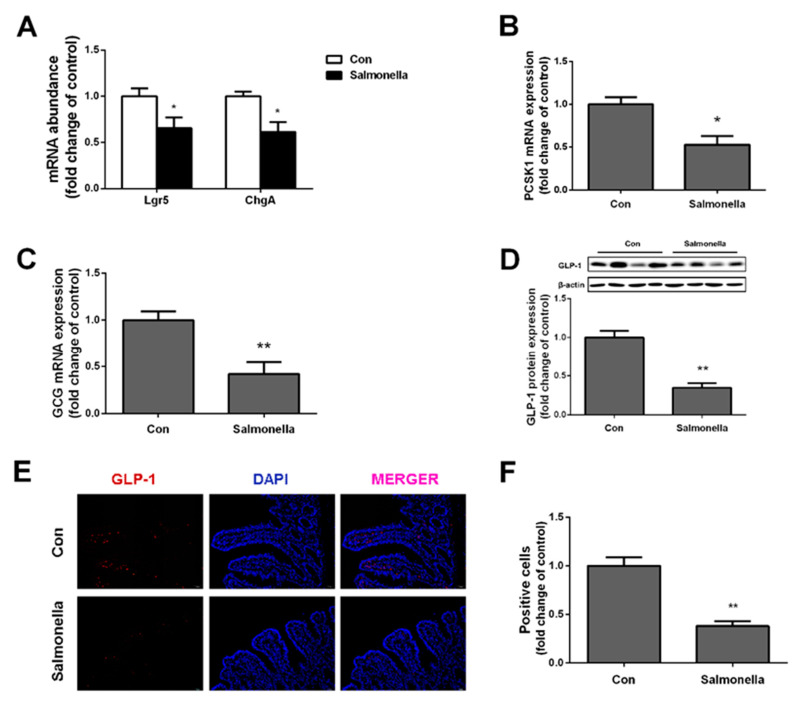
Effects of *Salmonella* on GLP-1 secretion in ileum. (**A**) gene expressions of cell type markers: Lgr5 (stem cells) and ChgA (endocrine cells); (**B**) PCSK1 mRNA expression; (**C**) GCG mRNA expression; (**D**) protein expression of GLP-1; (**E**) ileum immunohistochemistry (10×); (**F**) percentage of GLP-1 fluorescent area as positive cells marker. Values are mean ± SEM. *n* = 4/group. (*) means *p* < 0.05, (**) means *p* < 0.01.

We analyzed the expressions of leucine-rich repeat-containing G protein coupled receptor (Lgr5) and chromogranin A (ChgA); they represent stem cells responsible for the ongoing renewal of intestinal epithelium and endocrine cells, respectively. It was shown that Lgr5 and ChgA expressions were markedly decreased in the *Salmonella* infection group (Figure 2A). In the ilea of pigs treated with *Salmonella*, we found significant reductions of proprotein convertase subtilisin/kexin type 1 (PCSK1) and preproglucagon (GCG) gene expressions (Figure 2B,C), which are, respectively, responsible for post-translational processing of proglucagon and encoding the protein of GLP-1. Western blotting results revealed that *Salmonella* treatment significantly decreased protein expressions of GLP-1 (Figure 2D). Consistently, immunofluorescence staining analysis revealed that the secretion of GLP-1 was dramatically reduced in the *Salmonella* treatment group compared with the control group pigs (Figure 2E,F).

### 2.4. Effects of Salmonella Infection on Pyroptosis Signal Pathway in Ileum

**Figure 3:**
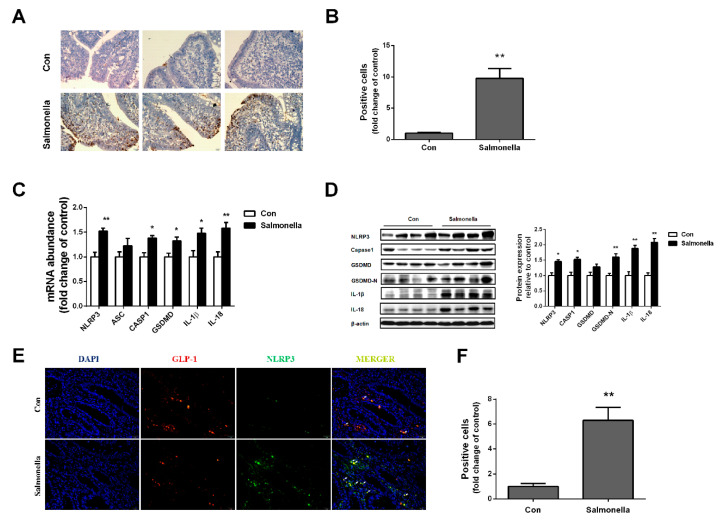
Effects of Salmonella on pyroptosis signal pathway in ileum. (**A**) TUNEL analysis; (**B**) positive cells for TUNEL analysis; (**C**,**D**) pyroptosis-related genes and protein expressions in ileum, respectively; (**E**) ileum immunohistochemistry (20×); (**F**) immunohistochemistry positive cells. Values are mean ± SEM, *n* = 4/group. (*) means *p* < 0.05, (**) means *p* < 0.01.

TUNEL examination was performed, as shown in Figure 3A,B. Compared with the control group, *Salmonella* treatment significantly increased the number of positive cells in the ileum. Then, we examined mRNA and protein levels of pyroptosis-related genes in ilea. As shown in Figure 3C, mRNA levels of NLRP3, CASPASE1, GSDMD, IL-1β, and IL-18 were dramatically up-regulated in the *Salmonella*-treated group when compared with the control group, although no significant difference was observed in ASC mRNA expression. Accordingly, Western blotting analysis revealed that the protein expressions of NLRP3, CASPASE1, GSDMD-N, IL-1β, and IL-18 were higher in ilea of the *Salmonella* treatment group than the control group (Figure 3D). To further explore whether *Salmonella* treatment induced pyroptosis of L cells in the ileum, we labeled L cells using both GLP-1 and NLRP3 fluorescent antibodies. After *Salmonella* treatment, the luciferase activity of GLP-1 significantly decreased, while NLRP3 luciferase activity significantly increased (Figure 3E,F).

### 2.5. Effects of Salmonella Infection on GLP-1R and Glucose Metabolism in Liver and Muscle

**Figure 4:**
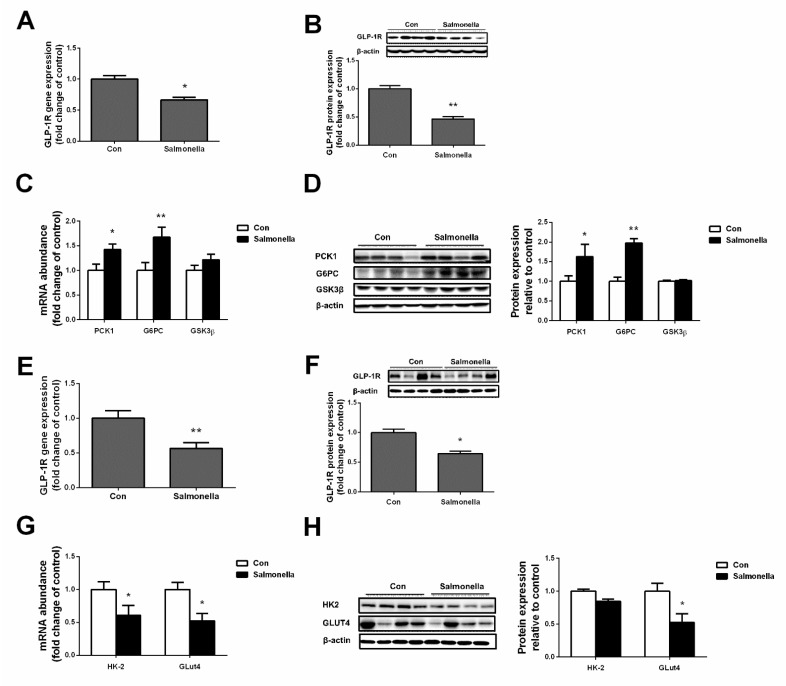
Effects of *Salmonella* on GLP-1R and glucose metabolism in liver and muscle. (**A**,**B**) genes and protein expression of GLP-1R in liver; (**C**,**D**) genes and protein expression of gluconeogenesis in liver; (**E**,**F**) genes and protein expression of GLP-1R in LGD; (**G**,**H**) genes and protein expression of glycolysis in LGD. LGD—longissimus dorsi. Values are mean ± SEM. *n* = 4/group. (*) means *p* < 0.05, (**) means *p* < 0.01.

**Figure 5:**
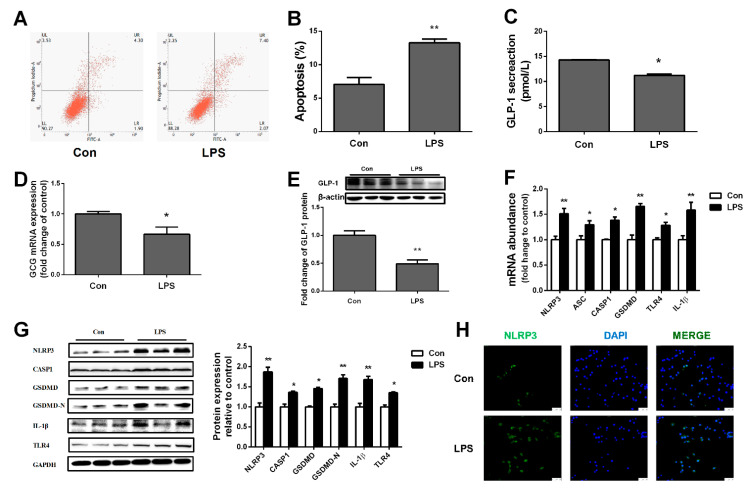
LPS inhibited production of GLP-1 by inducing pyroptosis in STC-1 cells. STC-1 cells were treated with LPS for 24 h. (**A**,**B**) apoptosis of STC-1 cells in control and LPS group was detected by flow cytometry assay; (**C**) GLP-1 content in STC-1 cells; (**D**) proglucagon mRNA expression; (**E**) protein expression of GLP-1; (**F**,**G**) pyroptosis-related genes and protein expression in STC-1 cells; (**H**) ileum immunohistochemistry (10×). Values are mean ± SEM. *n* = 3/group. (*) means *p* < 0.05, (**) means *p* < 0.01.

After *Salmonella* treatment, mRNA expressions of GLP-1R were significantly lower than the control group both in liver and longissimus dorsi (Figure 4A,E). Correspondingly, the protein expressions of GLP-1 were also dramatically reduced in the *Salmonella* group compared with the control group both in liver and longissimus dorsi (Figure 4B,F). Then, we performed gene expressions of gluconeogenesis in liver and glycolysis in longissimus dorsi. No significant changes were obtained for glycogen synthase kinase 3 beta (GSK3β) mRNA and protein levels between the control and *Salmonella* groups (Figure 4C and 5D). Conversely, the mRNA and protein levels of phosphoenolpyruvate carboxykinase 1 (PCK1) and glucose-6-phosphatase catalytic (G6PC) were up-regulated (*p* < 0.05) in the *Salmonella* group (Figure 4C,D). Meanwhile, mRNA and protein expressions of hexokinase-2 (HK2) and glucose transporters 4 (GLUT4) were obviously down-regulated (*p* < 0.05) in the longissimus dorsi of the *Salmonella* infection group (Figure 4G,H).

### 2.6. LPS inhibited the Production of GLP-1 by Inducing Pyroptosis in STC-1 Cells

LPS is the main component of the cell wall of Gram-negative bacteria. We used LPS-cultured STC-1 cells to simulate *Salmonella* infection. Flow cytometry showed that LPS increased STC-1 cell apoptosis (Figure 5A,B) and reduced the production of GLP-1 (Figure 5C). In addition, the levels of proglucagon mRNA and GLP-1 protein were dramatically decreased by *Salmonella* treatment (Figure 5D,E). To verify whether LPS could cause L cell pyroptosis, we determined the expressions of NLRP3, CASP1, and GSDMD. The results showed they were significantly up-regulated in the *Salmonella* group (Figure 5F,G). This was further certified by immunofluorescence staining of NLRP3 in STC-1 cells (Figure 5H). The results also further confirmed that *Salmonella* could induce L cell pyroptosis in ilea and reduce the secretion of GLP-1.

## 3. Discussion

It has been established that inflammatory mediators have a role in mediating metabolic disturbances. Additional evidence suggested that hyperglycemia developed during immune response, and it was perceived that hyperglycemia may play a central role in sustaining active T cells [3]. In the present study, we found that *Salmonella* infection severely damaged ileal epithelial structure and reduced the production of GLP-1. Although there were no significant differences in triglyceride and total cholesterol, *Salmonella*-infected pigs exhibited metabolic dysbiosis with a substantial increase in blood glucose, and HOMA-IR was dramatically increased (there were no significant differences in these indexes between the two groups before challenged with *Salmonella*).

Disturbed intestinal function is often associated with damaged intestinal mucosa and barrier function causing inefficient nutrient transport and absorption in pigs [16]. *Salmonella* as a pathogenic enterobacteriaceae could invade the intestinal epithelium and increase the susceptibility of hosts relative to developing intestinal inflammation [17]. The completeness of the intestinal epithelial barrier plays a key role in the maintenance of intestinal tract homeostasis under stress conditions caused by bacteria, viruses, and environmental factors [18,19]. Previous studies found obvious injury to the intestinal epithelial barrier after *Salmonella* infection [20]. Here, our HE staining results showed that *Salmonella* infection resulted in the shedding of ileal epithelial cells, rupture of villi, and inflammatory infiltration. In previous research, oral infections with *Salmonella* significantly enhanced the mRNA levels of inflammation in gut and ileal mucosa [21,22]. Similar to our data, the expressions of TLR4 and p-NF-ΚB were up-regulated in piglets that suffered from *Salmonella*. In addition, TUNEL staining can show cell damage by marking the DNA that breaks during cell death; the TUNEL results of the present study further confirmed that *Salmonella* severely damaged ileal epithelial cells.

In glucose metabolic regulation research, GLP-1 is one of the most well-studied and characterized hormones [23,24]. GLP-1 is described as a potential target in the therapy of type 2 diabetes/obesity by means of repairing β-cell function and increasing insulin sensitivity in tissues [25]. However, studies on the damage to gut L cells induced by inflammation remain limited. In the current study, we detected the level of GLP-1 in plasma and found that *Salmonella*-challenged pigs showed lower content of GLP-1; meanwhile, a significant decrease in mRNA expressions of GCG and PCKS1 was observed. Furthermore, we found that the expressions of GLP-1R both in liver and muscle were dramatically down-regulated after *Salmonella* treatment, which may result in enhanced gluconeogenesis in liver and disturbed glycolysis in muscles. As we know, the effects of *Salmonella* infection on GLP-1 secretion have not been currently reported. This study’s results may provide a new target to study in the effects of *Salmonella* on glucose metabolism. Previous research found that downregulation of inflammatory responses in the intestine was positively associated with increased secretion of GLP-1 from the intestine [26]. In our results, *Salmonella* infection resulted in a decrease in piglet GLP-1 secretion and a decrease in insulin levels. These observations may be related to the mechanism of GLP-1 improving hyperglycemia by improving insulin secretion [24].

Pyroptosis, the other form of programmed necrosis, is emerging as a general innate immune effector mechanism in response to certain bacterial insults. It has been well characterized that the mechanism of macrophage pyroptosis is induced by *Salmonella* [27,28]. Pyroptosis could contribute to the development of autoimmune and inflammatory diseases by IL-1β/IL-1 [29]. *Salmonella* infection could induce apoptosis of intestinal epithelial cells [30]. Pyroptosis of intestinal endocrine L cells’ responses to *Salmonella* is less illustrated. In our results, protein expressions of IL-1β/IL-18 were significantly higher compared with the control group. Activations of pro-caspase-1, cleaved by inflammasomes, are known to be a crucial step for inflammasomes to mediate pyroptosis and IL-1β/IL-18 production. Meanwhile, inflammasomes require a priming signal induced by Toll-like receptors [31]. In ilea, *Salmonella* infection caused damage-induced intestinal cell death and NLRP3 activation in the present study. CASP1 were dramatically up-regulated both in mRNA and protein levels after *Salmonella* treatment. In addition, GSDMD was proven to be a key protein in the activation of pyroptosis [32]. The active N-terminal fragment oligomerizes to form membrane pores that cause leakage of pro-inflammatory cytokines [12]. Consistent with previous research, we found that *Salmonella* infection significantly enhanced both protein and mRNA expressions of GSDMD and the protein expression of GSDMD-N.

Meanwhile, we treated STC-1 cells in vitro (a type of enteroendocrine L cell) with LPS to simulate the intestinal L cell injury model caused by *Salmonella*. It has been shown that LPS inhibits the growth of STC-1 cells in a concentration-dependent manner [33]. Other researchers have found that LPS inhibits secretion of GLP-1 in L cells [34]. Consistent with previous studies and according to flow cytometry data in our research, the number of apoptosis cells was remarkably increased after treatment with LPS. Our study demonstrated that LPS could induce pyroptosis of STC-1 through the TLR4/NLRP3/GSDMD signal pathway. Immunofluorescence results showed that LPS administration significantly enhanced the fluorescence intensity of NLRP3 in STC-1 cells, further confirming that LPS could cause pyroptosis of STC-1 cells.

## 4. Materials and Methods

### 4.1. Animals

Twelve 5-week-old male piglets (Duroc × Landrace × Large White) were randomly divided into two groups (6 in each group): the normal (Con) group and the *Salmonella* infection *(Salmonella)* group. In the *Salmonella* group, bacterial strains (10^9^ CFU/day) were administered by oral gavage at the ages of 7 and 8 weeks (once a week). The control group was intragastric with normal saline. All piglets had ad libitum access to diet and water. At 9 weeks of age, all piglets from each group were slaughtered for collecting blood and tissue samples. In our experiment, all animal care procedures and experimental protocols were authorized by the Animal Welfare Committee of Nanjing Agricultural University (Nanjing, China) and were performed according to the Committee of Animal Research Institute guidelines.

### 4.2. Cell Culture

The mice secretin tumor cell line (STC-1) was purchased from Beijing Beina Chuanglian Biotechnology Institute (Beijing, China). The cells were cultured in DMEM (SH30243.01, Hyclone, Logan, UT, USA) containing 10% (*v*/*v*) FBS (A31608-02, Gibco, Carlsbad, CA, USA) and antibiotics (100 IU/mL penicillin and 100 IU/mL streptomycin) at 37 °C with 5% CO_2_. The medium was changed every 2 days. Cells were trypsinized with 0.25% trypsin-EDTA (Sigma-Aldrich, St. Louis, MO, USA) when they reached 80–90% confluence. Each experiment was repeated at least three times (*n* ≥ 3).

### 4.3. RNA Isolation and Quantitative Real-Time PCR

Total RNA was extracted from ileum, liver, muscle, and cell samples using 1 mL of TRIzol reagent (Invitrogen, Carlsbad, CA, USA). Up to 1 μg of the RNA samples was used for reverse-transcription according to the manufacturer’s information (Vazyme Biotech, Nanjing, China). cDNA (1:10) was used as a template in PCR reactions on a real-time PCR system (Mx3000P; Stratagene, LaJolla, CA, USA). GAPDH was used as a reference gene to normalize the mRNA abundance of target genes. The primers were provided by Generay Biotech and are listed in Table 2 and Table 3. Finally, we used the 2^−ΔΔCt^ method to analyze real-time PCR data.

### 4.4. Western Blot Analysis

Total protein was extracted from STC-1 cells, ileum, liver, and dorsal longissimus muscle samples using an RIPA buffer that contained a protease inhibitor (P8340, Sigma, St. Louis, MO, USA). The protein concentration was determined according to the manufacturer’s instructions of the Pierce BCA Protein Assay kit (Rockford, IL, USA). Protein was then used for electrophoresis on a 10% or 15% SDS-PAGE gel. Target proteins were blotted onto nitrocellulose membrane by transferring. Then, membranes were blocked in 4% (*v*/*v*) skimmed milk for 2 h at room temperature and then incubated with primary antibodies overnight at 4 °C. 1×TBS with 0.1% Tween 20 used for washing membranes (three times for 10 min each time). Membranes were incubated for 2 h in secondary antibodies and washed for another three times. Finally, images were captured by a VERSADOC MP 4000 system (Bio-Rad, California, CA, USA), and the band density was analyzed with Quantity One software (Bio-Rad, California, CA, USA). Antibodies against β-actin served as an endogenous reference. 

### 4.5. ELISA

The levels of GLP-1 (Sensitivity: 0.025 pmol/L), insulin (Sensitivity: 0.75 mIU/L), and glucagon (Sensitivity: 0.375 pg/mL) in plasma were measured by an ELISA kit (MEIMIAN, Jiangsu, China) according to the manufacturer’s instructions.

### 4.6. Immunofluorescence Analysis

Immunofluorescent staining was performed by using 5 μm thick sections from ileal tissues that were routinely fixed in formaldehyde and embedded into paraffin. In order to prepare the antigen, the sections were boiled with sodium citrate for 10 min. 1×TBST containing 0.3% Triton X 100 was used for permeabilization and blocked in 10% (*v*/*v*) bull serum albumin (BSA). Then, the sections were incubated overnight at 4 °C with primary antibodies mouse monoclonal anti-GLP-1 antibody (Santa Cruz Biotechnology Inc., Santa Cruz, CA, USA; SC-80602; 1:50) and rabbit polyclonal anti-NLRP3 antibody (Abcam, Cambridge, England; ab55543, 1:33). Before the sections were incubated with the appropriate secondary antibodies (anti-mouse IgG-Alexa 488 for anti-GLP-1, anti-rabbit IgG-Alexa 546 for NLRP3) for 1 h at 37 °C, they were first washed with 1×TBST (containing 0.3% Triton X-100) three times for 5 min each time. 4′,6-diamidino-2-phenylindole (DAPI) (Sigma-Aldrich, St. Louis, MO, USA, D9564) was used to stain the nucleus. Image capture and analysis were performed using AxioVision 4.8.2 software (Carl Zeiss, Munich, Germany). For cell immunofluorescent staining, 1 × 10^5^/well STC-1 cells were inoculated into 24-well plates for immunofluorescence analysis. When they reached 80% confluence, cells were washed three times with PBS (pH = 7.4). Afterward, cells were fixed in 4% PFA and permeabilized using 0.3% Triton X-100. The rest of the procedure was the same as tissue immunofluorescence methods. 

### 4.7. Histopathology of the Ileum

Ileal tissues with 5-micrometer-thick sections were used for hematoxylin-eosin (HE) staining or TdT-mediated dUTP Nick-End Labeling (TUNEL) apoptosis assays. In order to detect the severity of intestinal damage, HE staining was performed according to the directions provided by the manufacturer. A Colorimetric TUNEL Apoptosis Assay Kit (Beyotime Biotechnology, C1901, China) was used for apoptosis assays following the manufacturer’s instructions. Briefly, the sections were dewaxed to water, followed by 20 ug/mL proteinase K (Sigma, St. Louis, MO, USA) for 20 min at 37 °C. Three percent H_2_O_2_ was used to quench endogenous peroxidase immersed with 0.3% Triton X-100 for 10 min, followed by 10 mg/mL proteinase K (Sigma, St. Louis, MO, USA) for 20 min at room temperature. After that, the sections were incubated with reaction solution containing terminal deoxynucleotidyl transferase and fluorescein-labeled dUTP at 37 °C for 60 min in the dark. Finally, nuclei were stained with hematoxylin. Image capture and analysis were performed using AxioVision 4.8.2 software (Carl Zeiss, Munich, Germany).

### 4.8. Apoptosis Analysis Using Flow Cytometry

Briefly, STC-1 cells were cultured in six-well plates at a density of 5 × 10^5^ per well. Afterward, STC-1 cells were incubated with LPS at a concentration of 1 μg/mL. When cells reached 90–95% confluence, they were washed two times with PBS. Thereafter, an Annexin V–FITC Apoptosis Detection Kit (Medical & Biological Laboratories, Nagoya, Japan) was used to assess the apoptosis rate according to the manufacturer’s protocol.

### 4.9. Statistical Analysis

All data were statistically analyzed using SPSS 18.0 for Windows and were expressed as the mean ± standard error of mean (SEM). The data were analyzed by one-way ANOVA followed by Student’s *t*-test. When *p* < 0.05, data were considered statistically different.

## 5. Conclusions

**Figure 6:**
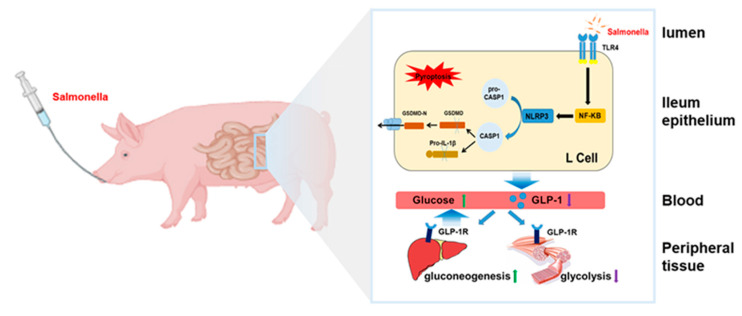
Schematic representation showing that *Salmonella* activation of NLRP3/GSDMD pathway causes pyroptosis of intestinal L cells, which reduces GLP-1 secretion and increases peripheral glucose levels.

In the present study, *Salmonella* exposure enhanced plasma inflammatory factor levels and caused damaged morphological structure with pyroptosis of ileum L cells, resulting in lower contents of GLP-1 in plasma. These changes may result in glucose metabolism disorder (Figure 6). *Salmonella* are Gram-negative bacterial pathogens that infect a range of hosts and cause several diseases. The results further illustrate evidence for hyperglycemia induced by inflammation and will be useful for developing new therapeutic targets for hyperglycemia prevention and treatment.

## Figures and Tables

**Table 1:** Effects of *Salmonella* on the blood parameters of piglets.

Parameters	Con	*Salmonella*	*p*-Value
TNFα (pg/mL)	10.17 ± 1.76	35.26 ± 2.49	0.007
IL-10 (pg/mL)	286.43 ± 25.72	196.91 ± 7.95	0.032
FBG (mmol/L)	2.46 ± 0.36	4.59 ± 0.29	0.002
FINS (mIU/L)	13.82 ± 0.95	11.13 ± 0.88	0.039
GLP-1 (pmol/L)	12.74 ± 0.58	9.54 ± 0.49	0.009
HOMA-IR	1.47 ± 0.11	2.36 ± 0.27	0.023

TNFα—tumor necrosis factor α; IL-10—interleukin 10; FBG—fasting blood glucose; FINS—fasting blood insulin; HOMA-IR—homeostasis model assessment insulin resistant. Results are shown as means ± SEM (*n* = 6).

**Table 2:** Primers of mice for real-time PCR.

Target Genes	GenBank Accession Number	Primer Sequences
β-actin	NM_007393	F:5′-CCACCATGTACCCAGGCATT-3′ R:5′-CAGCTCAGTAACAGTCCGCC-3′
GCG	NM_008100.4	F:5′-TCTACACCTGTTCGCAGCTC-3′ R:5′-TCCTCATGCGCTTCTGTCTG-3′
NLRP3	NM_001359638.1	F:5′-AGCCAGAGTGGAATGACACG-3′ R:5′-CGTGTAGCGACTGTTGAGGT-3′
ASC	NM_023258.4	F:5′-AACTGCGAGAAGGCTATGGG-3′ R:5′-CTGGCTGTACTCTGAGCAGG-3′
Caspase1	NM_009807.2	F:5′-GGACTGACTGGGACCCTCAA-3′ R:5′-TACCCCAGATCCTCCAGCAG-3′
GSDMD	XM_006521343	F:5′-GGGGATGACCTGTTTGTGGT-3′ R:5′-ATAAGCAGTTGGGCCACTCG-3′
TLR4	XM_036163964.1	F:5′-CCACAAGAGCCGGAAGGTTA-3′ R:5′-CAGAAGATGTGCCTCCCCAG-3′
IL-1β	NM_008361.4	F:5′-GTGCAAGTGTCTGAAGCAGC-3′ R:5′-CAAAGGTTTGGAAGCAGCCC-3′

**Table 3:** Primers of pig for real-time PCR.

Target Genes	GenBank Accession Number	Primer Sequences
GAPDH	NM_001206359.1	F:5′-GTATGATTCCACCCACGGCA-3′ R:5′-CACCCCATTTGATGTTGGCG-3′
GCG	NM_214324	F:5′-GTTAATCCAGACAGCCATGCG-3′ R:5′-TGACCTGAGGACTGCTTTCAC-3′
PCSK1	NM_214038	F:5′-GTTGCGAAGGTAGCGG-3′ R:5′-CAATGGCAGAGGATGGTA-3′
G6PC	NM_001113445.1	F:5′-GTGATCGCGGACCTCAGAAA-3′ R: 5′-AGCCAGTCTCCAATCACAGC-3′
GSK3β	NM_001128443	F:5′-CTGCCCAAGGCAGAGAAACT-3′ R: 5′-ATTCAGATTGCCCAAGCCGT-3′
PCK1	NM_001123158.1	F: 5′-CCTCCTCAGCTCTCAAACGG-3′ R: 5′-AGCCAACCAGCAGTTGTCAT-3′
HK2	NM_001122987	F:5′-GACTCAAGACGAGGGGCATC-3′ R: 5′-CGTCACTTTGAGGGTGTCCA-3′
GLUT4	NM_001128433	F: 5′-TGGACGGTTCCTCATTGGTG-3′ R: 5′-GTGATGCCCAGGAGTAGTGG-3′
NF-ΚB	NM_001279309	F:5′-ACCATTGCTAAACTCCGCCA-3′ R:5′-GAATGGTGGAGTGCTTCCCT-3′
NLRP3	NM_001256770	F:5′-TCGGGGCCAGACAGAAAAAG-3′ R:5′- CCCATTCTGGCTCATCCCTC-3′
ASC	XM_003124468	F:5′-AGCAGACAACAAACCAGCAC-3′ R:5′-TCCACGTCTGTGACCCTTGA-3′
Caspase1	NM_001043585.1	F:5′-AACGGCAATGAAGACGAAGG -3′ R:5′-ATTTTCGCATAACGTCGCCC -3′
GSDMD	XM_021090506.1	F:5′-GGAACGATGTGTTCGTGGTG -3′ R:5′-CCCGAAATGCAATGACGCTG -3′
IL-1β	NM_214055.1	F:5′-AGGCTGGAGAAAAAGCAGCA-3′ R:5′-CCCGGTACAGATGGCAAGTT-3′
IL-18	XM_005667326.2	F:5′-TCCGGATCACTTCCTCTCGT-3′ R:5′-CCGATTCCAGGTCTTCATCGT-3′
Lgr5	XM_021090898.1	F:5′-TCTGGTGATTGTCCCGCTTC-3′ R:5′-CTTGGGTGGAGTCACAGGAC-3′
ChgA	NM_001164005	F:5′-ACTCCGAGGAGATGAACGGA-3′ R:5′-GCGAGGTCTTGGAGCTCTTT-3′

## Data Availability

Data are contained within the article.
